# The mitochondrial chaperone HSPD1 folds MTHFD2 independently of its co-chaperone HSPE1

**DOI:** 10.1098/rstb.2025.0500

**Published:** 2026-10-01

**Authors:** Shani Gabbay, Hila Ben-David, Shatha S. Alassam, Linor Cohen, Tal Levy, Albert Batushansky, Shiran Dror, Moshe Elkabets, Noga Alon, Shai Kaluski-Kopach, Shimon Bershtein, Anat Ben-Zvi, Barak Rotblat

**Affiliations:** ^1^ Department of Life Sciences, Ben-Gurion University of the Negev, Beer-Sheva, Israel; ^2^ Ilse Katz Institute for NanoscaleScience & Technology, Ben-Gurion University of the Negev, Beer-Sheva, Israel; ^3^ The Shraga Segal Department of Microbiology, Immunology, and Genetics, Ben-Gurion University of the Negev, Beer-Sheva, Israel

**Keywords:** mitochondria, MTHFD2, cancer, one-carbon, chaperones, HSPD1

## Abstract

Acquiring new cellular states entails metabolic reprogramming driven by changes in the expression of cytosolic and mitochondrial metabolic enzymes. Most mitochondrial proteins are synthesized in the cytosol and imported into the mitochondria in a linear form, after which they are folded by a network of mitochondrial chaperones and co-chaperones. Which mitochondrial protein is dependent upon which chaperone for its folding is largely unknown. HSPD1/HSPE1 (HSP60/HSP10) are evolutionarily conserved mammalian homologues of the bacterial proteins GroEL/GroES, forming a chamber-and-lid chaperonin to facilitate the folding of client proteins. The endogenous clients of HSP60 in mammalian cells are not fully known, nor are the HSP60 clients that require HSP10 for folding. We used gene knockdown and stable isotope labelling of amino acids in cell culture (SILAC)-based proteomics to identify HSPD1 client proteins. We found that HSPD1 supports the expression of methylenetetrahydrofolate dehydrogenase 2 (MTHFD2), a key mitochondrial one-carbon (1C) pathway enzyme, in cells and tumours. In addition, HSPD1 directly folds MTHFD2 independently of its co-chaperone HSPE1. HSPD1 interacts with MTHFD2 in mitochondria, and MTHFD2 is degraded by LONP1 in HSPD1 knockdown cells. Our data show that HSPD1 is an MTHFD2 chaperone and can fold an endogenous client protein independently of HSPE1, providing a link between mitochondrial protein folding and the 1C pathway.

This article is part of the Theo Murphy meeting issue ‘ProteostaSys: a systems view of proteostasis’.

## 1. Introduction

Metabolic reprogramming is the process by which a cell changes its metabolic profile, such as during differentiation [1], immunity [1,2] and response to environmental cues [3]. In addition, metabolic reprogramming is a hallmark of cancer and a crucial aspect of the process by which a normal cell becomes malignant and metastatic [4–7]. Metabolic reprogramming entails the up- and downregulation of enzymes in the cytosol and mitochondria [8,9]. Most mitochondrial enzymes are encoded in the nuclear genome and synthesized in the cytosol. These enzymes are imported into the mitochondria in an unfolded state and must be correctly folded by mitochondrial chaperones [10]. Mitochondrial chaperones operate within sophisticated networks alongside other chaperones and co-chaperones to facilitate protein folding. Additionally, they fold stress-denatured mitochondrial proteins. When the stoichiometric balance between chaperones and their client proteins is disrupted, the consequences include protein misfolding, aggregation or premature degradation [11].

There is accumulating evidence that specific proteins depend on specific chaperones for folding. For example, the mitochondrial chaperone, TRAP1, stabilizes the electron transport chain complex II subunit succinate dehydrogenase-B, along with other mitochondrial proteins, to facilitate respiration during nutrient stress, supporting the possibility that specific mitochondrial chaperones are critical for specific mitochondrial functions [12]. In line with this concept, the expression of specific chaperones and their clients is coordinated in tissues during development, exemplified by upregulation of the myosin chaperone Unc45B during muscle development in *Caenorhabditis elegans*, indicating that molecular mechanisms have evolved to ensure sufficient chaperone capacity to accommodate the folding of proteins expressed in the particular biological context [13]. Using gene expression data from patient-derived tumours, we previously found that mitochondrial chaperones and proteins form a network comprised two major clusters [14,15], suggesting that coordinated expression of proteins with their chaperones evolved to provide sufficient chaperone expression to support the folding of these proteins.

HSPD1 and HSPE1 (HSP60 and HSP10) are chaperone-co-chaperone complexes, homologues of bacterial GroEL-GroES, together forming a protein folding chaperonin [16]. HSPD1 and HSPE1 reside in the mitochondrial matrix, assisting the folding of newly imported and synthesized proteins, as well as stress-induced misfolded proteins. The HSPD1 interactome was studied using crosslinking and immunoprecipitation, and more than 300 proteins were identified in complex with HSPD1, raising the question of which of these depend on HSPD1 for their folding in cells [17]. Treating cells with the small-molecule HSPD1 inhibitor KHS101 led to the aggregation of mitochondrial proteins involved in glycolysis, the tricarboxylic acid cycle, oxidative phosphorylation (OXPHOS) and mitochondrial quality control [18]. In addition, KHS101 reduced tumorigenicity in glioblastoma and lung cancer tumour cells *in vitro* and *in vivo* [19]. Furthermore, HSPD1 depletion led to reduced proliferation and oxygen consumption in lung cancer cells [19]. Conversely, HSPD1 knockdown (KD) in ovarian tumour cells resulted in increased proliferation by promoting the expression of the fatty acid elongation enzyme 3-oxoacyl-ACP synthase (OXSM) [20], implicating HSPD1 in fat metabolism. Recently, HSPD1, together with its co-chaperone HSPE1, was found to play essential roles in supporting the expression of mitochondrial ribosomal proteins and, therefore, for mitochondrial translation [21]. Nevertheless, it is not clear which HSPD1-dependent proteins are indeed HSPD1 clients.

To classify a protein as an endogenous chaperone client, three criteria must be satisfied: first, the protein must exhibit a physical interaction with the chaperone in cells; second, the protein's stability or solubility must be functionally dependent on the chaperone; and third, the chaperone must be shown to directly facilitate the folding of the client protein. The endogenous clients of HSPD1 and their dependence on HSPE1 are not well defined.

Here, we found that HSPD1 interacts with the mitochondrial one-carbon (1C) enzyme methylenetetrahydrofolate dehydrogenase 2 (MTHFD2) *in vivo*. HSPD1 KD leads to LONP1-dependent MTHFD2 degradation in tumour cells and HSPD1 directly folds MTHFD2 independently of HSPE1, establishing MTHFD2 as a *bona fide* HSPD1 client that does not require HSPE1 and linking HSPD1 to the 1C pathway.

## 2. Results

### (a) HSPD1-dependent mitochondrial proteins

We hypothesized that a subset of mitochondrial proteins depends on HSPD1 for folding. We reasoned that HSPD1 depletion would lead to aggregation or degradation of such HSPD1-client proteins. To test our hypothesis and identify HSPD1-dependent proteins, we attempted to generate HSPD1 knockout U251 cells, in which HSPD1 was found to be functional [18], using CRISPR. HSPD1 is an essential gene [22], which may explain why we failed to obtain single-cell-derived clones with complete ablation of HSPD1. To generate cells where HSPD1 is depleted by more than 80%, we chose clones with reduced HSPD1 expression and used lentivirus to stably express small hairpin RNA (shRNA) targeting HSPD1 (two independent sequences). Cells expressing scrambled shRNA (shCONT) were our controls (figure 1A).

**Figure 1.**
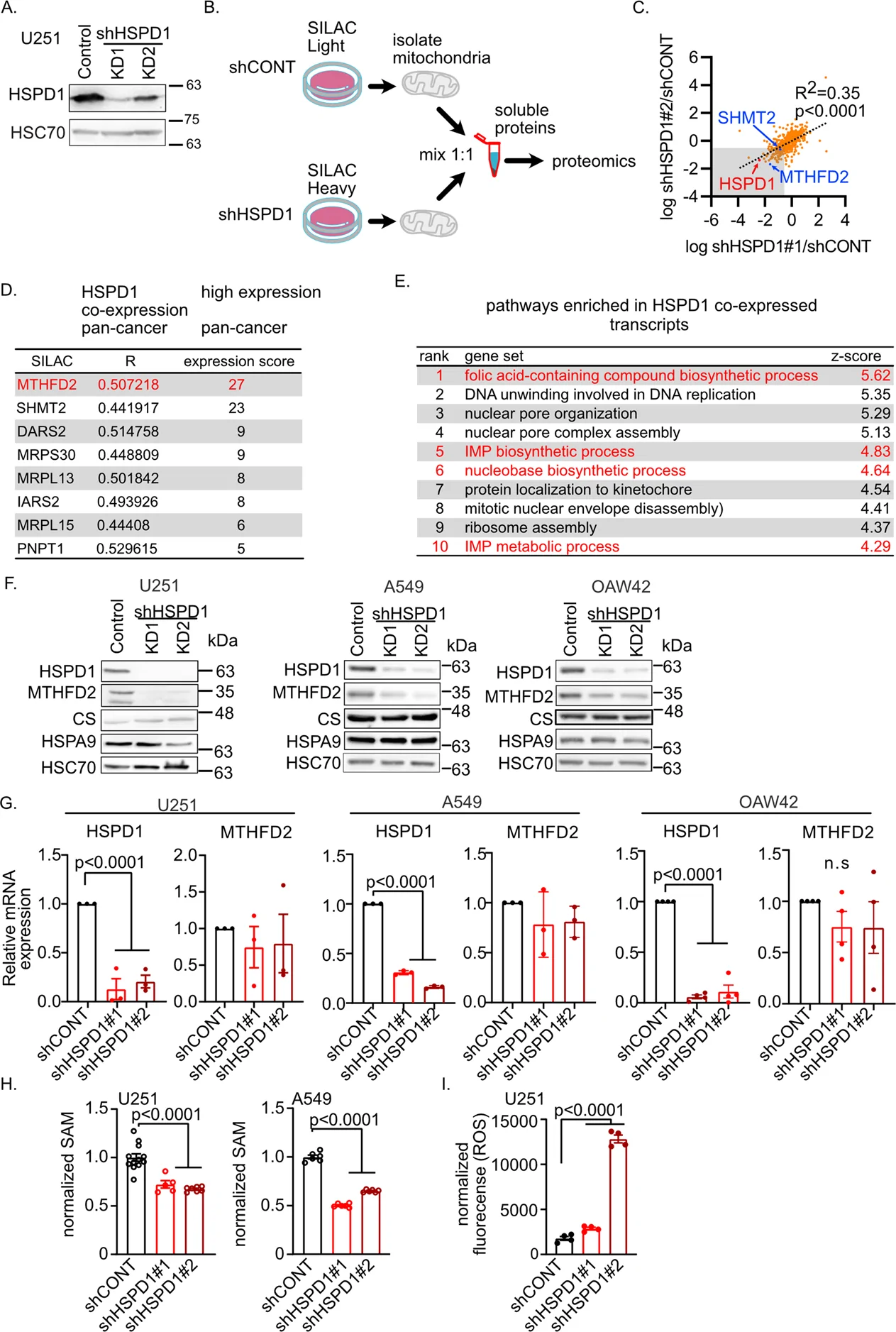
HSPD1 KD impacts MTHFD2. (A) Lysates of HSPD1 KD and control cells were analysed by a Western blot. HSC70 was used as a loading control. (B) Scheme depicting the stable isotope labelling of amino acids in cell culture (SILAC) experiment. (C) SILAC ratio of mitochondrial proteins in each HSPD1 KD versus control cells are plotted. The grey shaded area depicts proteins down-regulated by more than 50% in both KD cells versus the control. Mitochondrial one-carbon enzymes are depicted in blue. (D) Proteins ranked according to their co-expression with HSPD1 and expression level in different cancer types. (E) ARCHS4 analysis of enriched pathways in transcripts co-expressed with HSPD1 across multiple RNAseq datasets. (F) Lysates from HSPD1 KD and control cells were analysed by Western blot. HSC70 was used as a loading control. (G) Quantitative real-time polymerase chain reaction (qRT-PCR) was used to measure the expression of the indicated transcripts in HSPD1 KD and control cells. (H) S-adenosylmethionine (SAM) levels in HSPD1 KD and control cells. (I) Reactive oxygen species (ROS) levels in the indicated U251 cell lines were determined using dichlorodihydrofluorescein diacetate and fluorescence-activated cell sorting.

We took a proteomics approach to identify mitochondrial proteins that exhibit reduced solubility or expression in HSPD1 KD cells, as is expected in cases that depend on HSPD1 for their folding. We used stable isotope labelling of amino acirds in cell culture (SILAC) and labelled proteins of HSPD1 KD cells with heavy lysine and arginine and proteins of control cells with normal amino acids. We isolated mitochondria from cell lysates using anti-TOM22 beads (MACS), lysed the mitochondria and mixed the soluble fractions in a 1 : 1 ratio for proteomics analysis (figure 1B). We found 107 proteins whose expression was reduced in the two KD cell lines using -log2 < 0.5 as a cut-off (figure 1C; supplementary material, table S1). We interrogated the SILAC list of downregulated proteins using Enricher [23–25] and found enrichment for transfer RNA (tRNA) aminoacylation and 1C metabolism pathway in Reactome pathways and WikiPathways 2024 human, respectively (supplementary material, table S2).

Our model predicts that folding might be a limiting factor in cases where the chaperone's client is highly expressed, burdening the client's chaperones, and, therefore, the expression of the client and chaperone will be coordinated. To prioritize proteins for further analysis, we chose proteins whose expression is highly correlated with HSPD1 and is highly expressed in multiple cancer types. To rank proteins according to their expression levels in multiple cancer types and their co-expression with HSPD1, we used previously published analyses of metabolic enzyme expression in cancer [26] and mitochondrial chaperone-protein co-expression in multiple cancer types [14] (figure 1D). MTHFD2 was chosen for validation as it exhibited high co-expression with HSPD1 (ranked no. 3) and is highly expressed in multiple cancer types (no. 1). In addition, using the ARCHS4 database to interrogate more than 100 000 RNA-seq experiments [27], we found that HSPD1 is mostly co-expressed with the 1C pathway proteins (figure 1E), supporting the possibility that HSPD1 promotes the pathway.

MTHFD2 is a key mitochondrial enzyme in the 1C pathway. The mitochondrial 1C pathway provides carbons for nucleotide synthesis [28,29], S-adenosylmethionine (SAM) generation [30], and reducing power for antioxidants (NADPH)34, making it a promising drug target in oncology [31]. We identified another key mitochondrial 1C enzyme, SHMT2, as downregulated in our SILAC experiment (figure 1C). SHMT2 and MTHFD2 work in concert within the mitochondrial 1C pathway. SHMT2 generates 5,10-CH₂-THF, a substrate for MTHFD2. SHMT2 is co-expressed with HSPD1 (ranked no. 6) and is highly expressed in cancer (no. 2) (figure 1D).

Using Western blot, we confirmed reduced expression of MTHFD2 in HSPD1 KD cells and sustained expression of citrate synthetase (CS) and HSPA9, mitochondrial proteins that did not change significantly in our SILAC experiment (figure 1F). We validated MTHFD2 downregulation, but not CS or HSPA9, upon HSPD1 KD in two other cell lines (figure 1B) in which HSPD1 was found to be functional, A549 [19] and OAW42 [20].

The reduced expression of MTHFD2 protein in HSPD1 KD cells may result from decreased MTHFD2 messenger RNA (mRNA) levels in cells coping with reduced HSPD1 levels, rather than being because of MTHFD2 misfolding, aggregation and/or degradation. To rule this possibility out, we used RT-qPCR to measure HSPD1 and MTHFD2 mRNA levels in HSPD1 KD and control cells. We found reduced HSPD1 but not MTHFD2 mRNA levels in HSPD1 KD cells (figure 1G) and concluded that reduced MTHFD2 protein found in HSPD1 KD cells is not owing to reduced MTHFD2 mRNA levels. These results suggest that MTHFD2 is a client protein of HSPD1 and that HSPD1 may play an important role in the 1C pathway.

The 1C pathway is essential for the generation of a key methyl donor, SAM [32,33]. We therefore opted to measure SAM as a surrogate for the 1C output. We synthesized heavy SAM, spiked our lysis buffer with it, and used it as a standard to quantify SAM in HSPD1 KD and control cells. We found that, indeed, SAM levels are reduced in HSPD1 KD U251 cells and found that this is the case in A549 cells as well (figure 1H). The mitochondrial 1C in general and MTHFD2 in particular generate NADPH, a major cellular antioxidant, and MTHFD2 depletion leads to increased cellular reactive oxygen species (ROS; [34,35]). In agreement, we found increased ROS in HSPD1 KD cells (figure 1I). Together, these results support that HSPD1 interacts with the 1C pathway.

Since HSPD1 is an important chaperone and essential gene, our results using HSPD1 KD cells might reflect total mitochondrial collapse rather than misfolding of a specific subset of HSPD1 clients. To this end, we used Seahorse XF96 to measure mitochondrial respiration in HSPD1 KD and control cells (supplementary material, figure S1a,b). We did not find consistent differences in basal or maximal mitochondrial respiration following HSPD1 KD in U251 cells (supplementary material, figure S1a); however, HSPD1 KD did reduce basal and maximal mitochondrial respiration in A549 cells (supplementary material, figure S1b). In addition, we measured mitochondrial mass using MitoTracker Green and found that HSPD1 KD did not affect mitochondrial mass in U251 or A549 cells (supplementary material, figure S1C). Since mitochondrial mass and respiration were intact in HSPD1 KD cells and since HSPD1 depletion led to reduced expression of MTHFD2 but not CS or HSPA9, we can conclude that mitochondria did not collapse during the time of our experiments, supporting that our positive SILAC hits represent putative HSPD1 client proteins.

### (b) HSPD1 and methylenetetrahydrofolate dehydrogenase 2 are in close proximity in mitochondria

We next hypothesize that HSPD1 folds MTHFD2 in the mitochondria. While co-immunoprecipitation experiments found that the two proteins interact in cell lysates [17], it is unclear whether they interact in intact cells. We used proximity ligation assay (PLA), which detects potential protein–protein interactions (less than 40 nm proximity) in fixed cells using anti-HSPD1 and MTHFD2 antibodies. We found a clear perinuclear PLA signal in control cells, which was reduced in HSPD1 KD cells (figure 2A). Using MitoTracker RED to stain mitochondria, we found PLA signal localized in the mitochondria (figure 2B), supporting that HSPD1 and MTHFD2 are in close proximity to each other, in the mitochondria.

**Figure 2.**
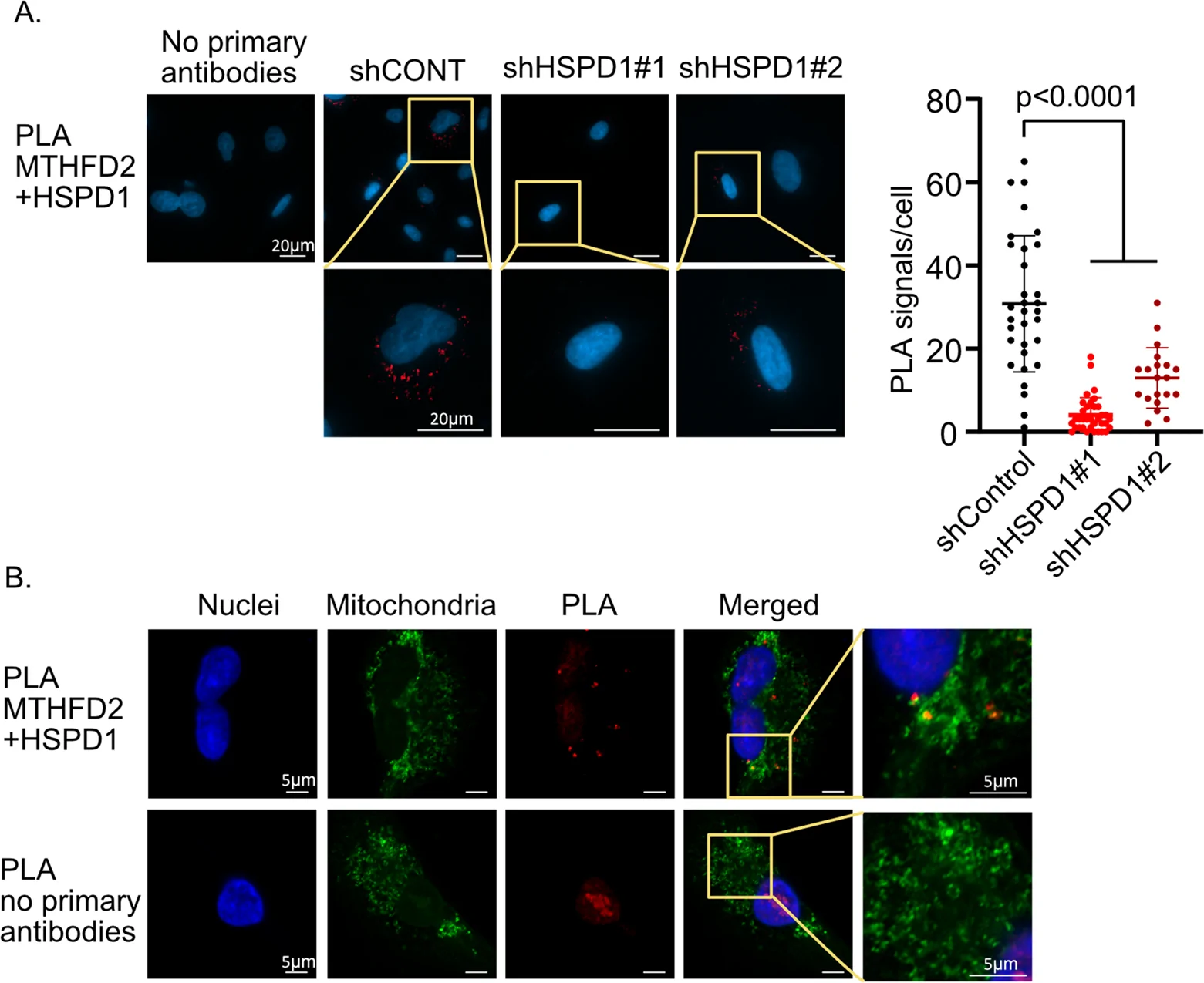
HSPD1 interacts with MTHD2 in mitochondria. (A) PLA analysis of HSPD1 and MTHFD2 in A549 cells was performed using HSPD1 KD cells and no primary antibodies as controls. Dots were counted in at least 40 cells. (B) PLA of HSPD1 interaction with MTHFD2 (red) and mitochondria staining (green).

### (c) A crosstalk between HSPD1 and methylenetetrahydrofolate dehydrogenase 2 in tumours

We used a mouse xenograft model to test whether HSPD1 facilitates MTHFD2 protein expression in tumours. HSPD1 KD and control A549 cells were injected into the flanks of NOD-SCID mice. Tumours were excised after 38 days, mass measured (supplementary material, figure S3a) and the levels of HSPD1 and MTHFD2 were determined using Western blot (figure 3A; supplementary material, figure S3b). There were no differences in tumour size, suggesting that HSPD1 is not essential for A549 tumourigenicity. We found reduced HSPD1 and MTHFD2 in tumours generated by HSPD1 KD cells (figure 3A; supplementary material, figure S3a), indicating that it facilitates MTHFD2 protein expression in tumours.

**Figure 3.**
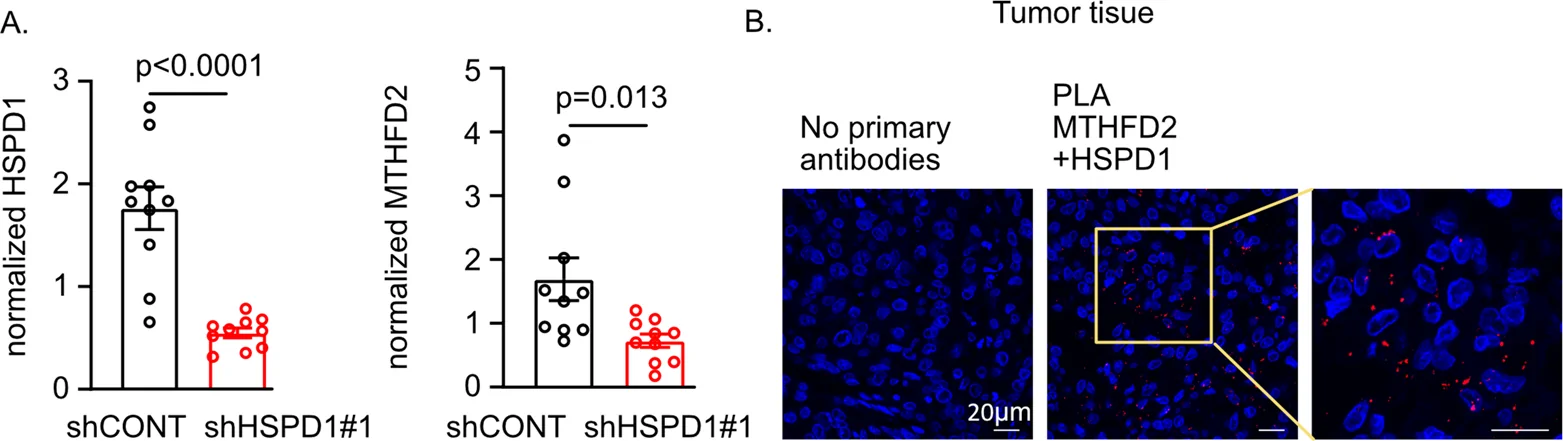
HSPD1 facilitates MTHFD2 protein expression in tumours. (A) HSPD1 and MTHFD2 levels in tumours generated by HSPD1 KD and control A549 cells were measured using Western blot and normalized to loading control. (B) PLA between HSPD1 and MTHFD2 in A549 tumours generated by shCONT cells.

To determine if HSPD1 and MTHFD2 interact in tumour tissue, we used PLA and shCONT tumour tissue. We found a clear PLA signal in the stained tissue (figure 3B), indicating that HSPD1 and MTHFD2 interact, or are at least in close proximity (less than 40 nm) to HSPD1 in tumour tissue. We conclude that HSPD1 interacts with MTHFD2 in tumour tissue, thereby supporting its expression.

### (d) Methylenetetrahydrofolate dehydrogenase 2 is degraded by LONP1 in HSPD1 KD cells

We asked if increased protein degradation rates can explain the reduced MTHFD2 levels found in HSPD1 KD cells. We pulsed cells with cyclohexamide (CHX) to stop new protein synthesis and measured the levels of remaining MTHFD2 at different time points following treatment (figure 4A). We calculated MTHFD2 half-life using densitometry measurements of band intensities and plotting the ln values over time. We found reduced MTHFD2 half-life in HSPD1 KD cells, indicating increased MTHFD2 degradation rates in HSPD1 KD cells.

**Figure 4.**
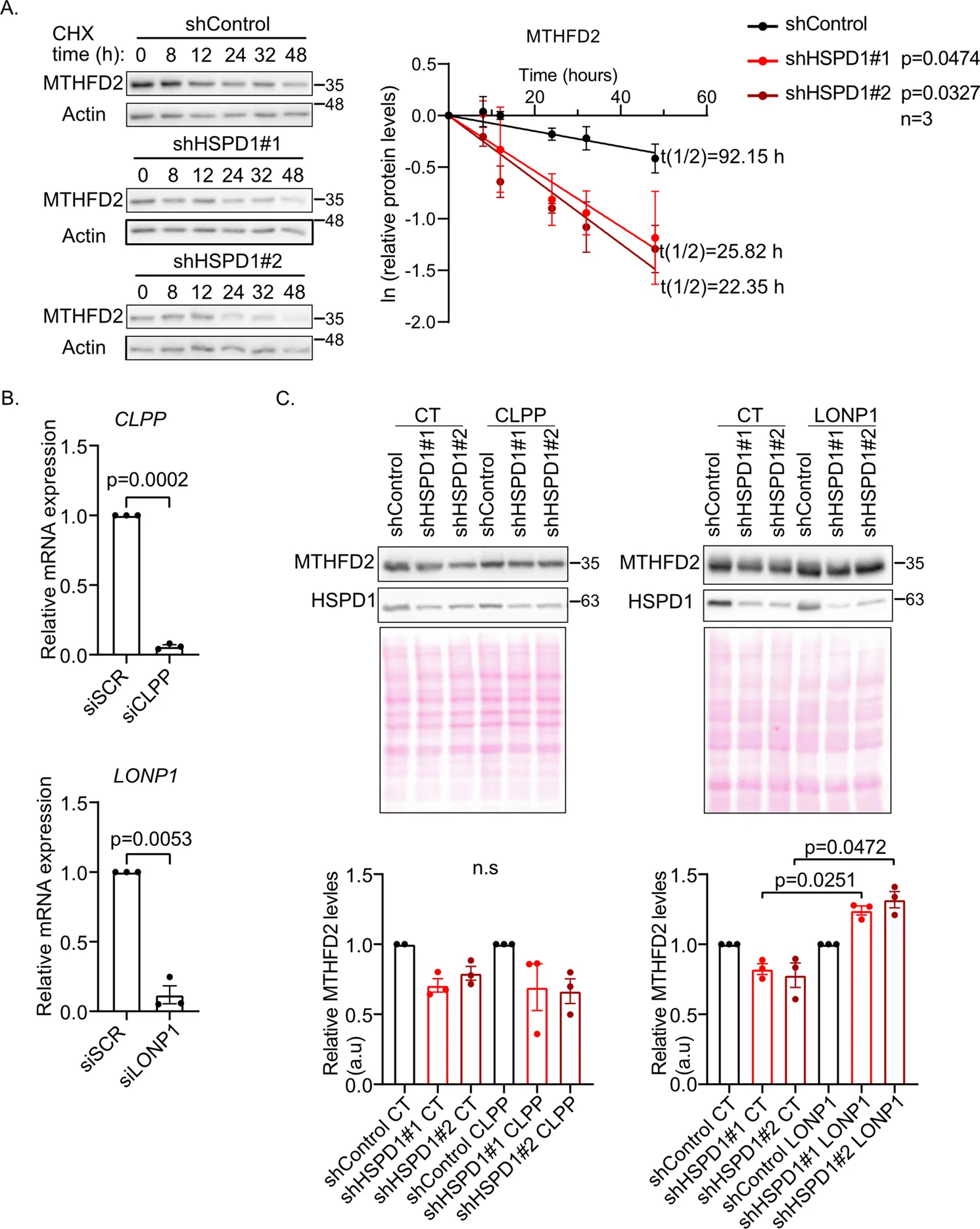
MTHFD2 is degraded by LONP1 in HSPD1 KD cells. (A) Cells were treated with CHX for the indicated time points, after which they were lysed and analysed by Western blot. Actin was used as a loading control. MTHFD2 levels were normalized to actin and time point 0. (B) Pooled small interfering RNA were used to KD CLPP and LONP1. KD was validated using qRT-PCR. *n* = 3. (C) MTHFD2 levels in siCLPP and siLONP1 in HSPD1 KD and control cells were measured using Western blot. Ponceau staining was used as a loading control. Significance was measured in siCLPP and siLONP1 versus siCONTROL in HSPD1 KD cells. *n* = 3.

Mitochondria are equipped with quality control mechanisms. The CLPP and LONP1 proteases have been shown to eliminate misfolded proteins in the mitochondrial matrix [36]. We used small interfering RNA to KD CLPP and LONP1 (figure 4B) and used Western blot to measure MTHFD2 in HSPD1 KD and control cells (figure 4C). LONP1 KD, but not CLPP1 KD, restored MTHFD2 levels in HSPD1 KD cells (figure 4D). These results support that MTHFD2 is degraded by LONP1 in HSPD1 KD cells and are in accord with recent data demonstrating that in HSPD1 KD cells, LONP1 degrades aggregation-prone mitochondrial proteins [21].

### (e) HSPD1 folds methylenetetrahydrofolate dehydrogenase 2 independently of HSPE1

To test whether HSPD1 physically interacts with and folds MTHFD2, we used recombinant MTHFD2 and HSPD1 and a chaperone-assisted folding assay (figure 5A). We confirmed that our recombinant MTHFD2 binds CH2methylene-THF using thermal shift assay (supplementary material, figure S4a). As a surrogate for proper folding, we measured the enzymatic activity of recombinant MTHFD2 using the substrates methylenemethenyl-THF and NADPH and reading the accumulation of the products, methenylmethylene-THF and NAD, using a plate reader at optical density 350 nm (supplementary material, figure S4b).

**Figure 5.**
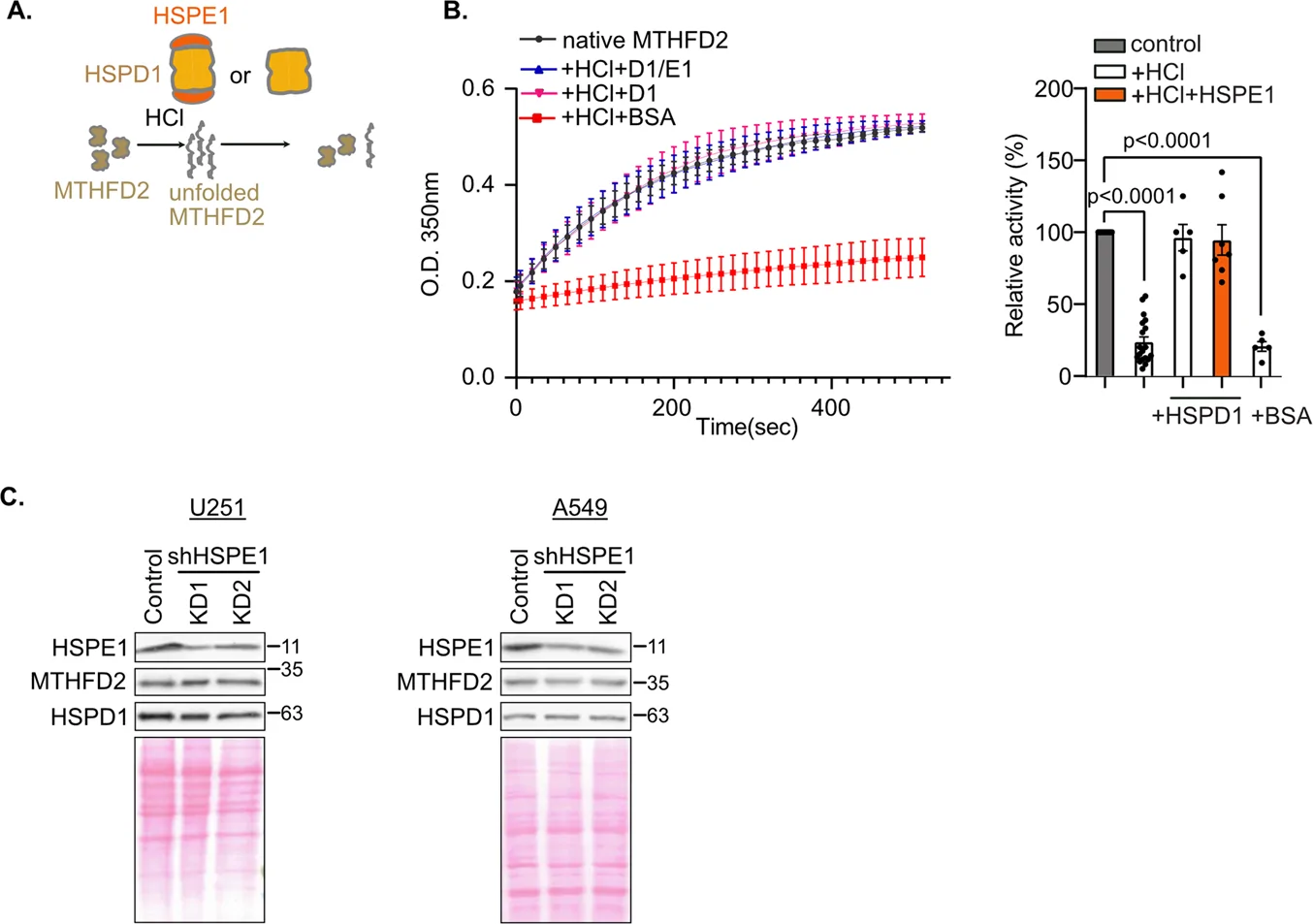
HSPD1 folds MTHFD2 independently of HSPE1. (A) Scheme depicting chaperone-assisted folding assay. (B) MTHFD2 activity following acid treatment was measured using a plate reader. The slope of the curve was used to determine MTHFD2 activity. Bovine serum albumin was used to control for crowding effect. (C) MTHFD2 levels in HSPE1 KD and control cells were measured using Western blot. Ponceau S staining was used as a loading control.

We treated MTHFD2 with acid to promote unfolding, restored pH to 7.2 and measured MTHFD2 enzymatic activity. We found acid eliminated MTHFD2 enzymatic activity, indicating misfolding. Following acid treatment and neutralization, we added HSPD1 and HSPE1 to the misfolded MTHFD2 and immediately measured MTHFD2 activity. We found that HSPD1 restored MTHFD2 to its fully active form (figure 5B). Surprisingly, HSPD1 could do so independently of its co-chaperone, HSPE1 (figure 5B). Interestingly, it was previously reported that the bacterial homologue of HSPD1, GroEL, folds misfolded bovine rhodanese independently of GroES, the bacterial homologue of HSPE1 [37].

Structural studies of the HSPD1–HSPE1 complex and their closely related bacteria homologues, GroEL–GroES, support that HSPE1 and GroES play key roles in HSPD1 and GroEL substrate folding activity [38]. Having found that HSPD1 folds MTHFD2 independently of HSPE1 *in vitro* (using recombinant proteins), we predicted that HSPD1 would be sufficient to support MTHFD2 expression in cells. We used shRNA to KD HSPE1 in A549 cells and measured MTHFD2 levels in HSPE1 KD and control cells (figure 5C). We found that HSPE1 depletion did not affect MTHFD2 or HSPD1 expression levels.

To identify the role of HSPE1 in mitochondrial respiration, we used the Seahorse XF96 and found that HSPE1 KD reduced basal and maximal respiration (supplementary material, figure S4c) but did not affect mitochondrial mass in A549 cells (supplementary material, figure S4d). These findings are in agreement with the findings in HSPD1 KD A549 cells and support that the reduced respiration in HSPD1 and HSPE1 KD cells may not be related to MTHFD2. Together, these results suggest that HSPE1 KD does not lead to total mitochondrial collapse and that HSPE1 does not play an important role in MTHFD2 folding.

## 3. Discussion

Using SILAC-based proteomics and HSPD1 KD cells, we found that MTHFD2 protein expression depends on HSPD1. We confirmed that MTHFD2 is an endogenous client of HSPD1 that does not require HSPE1. We will discuss the implications of these findings for understanding the role of HSPD1 in supporting cellular metabolism, the mitochondrial chaperone-client network and its HSPE1-independent molecular chaperone function.

Previously, HSP60 and HSP10 temperature-sensitive yeast mutants were used to identify HSP60 and HSP10 client proteins in isolated mitochondria [39]. Nine proteins were identified and validated as putative HSP60 or HSP10 clients. It is not known if these proteins bind HSP60 in cells or if HSP60 can fold them. HSPD1 mutants associated with human pathologies have been used to identify HSPD1 clients and to infer possible mechanisms linking HSPD1 mutations to the misfolding of HSPD1 substrates and diseases [5,40]. More than 400 putative clients were identified, 60 of which were identified in our SILAC experiment using HSPD1 KD cells, including the mitochondrial 1C enzyme MTHFD2 [5]. In support, analysis of multiple knockout mouse models experiencing severe OXPHOS dysfunction revealed upregulation of HSPD1 and MTHFD2 protein and transcript levels as a consistent and early response to mitochondrial stress [41]. Recently, HSPD1 was shown to play important roles in mitochondrial translation by promoting mitochondrial ribosomal proteins [21] and mitochondrial import proteins [42]. Nevertheless, whether the previously identified proteins are truly endogenous HSPD1 clients or whether HSPD1 can directly fold these proteins, with or without HSPE1, remains unknown. Here, we used a gene KD approach, degradation assays, and an *in vitro* chaperon-assisted folding assay to show that MTHFD2 is an endogenous HSPD1 client.

The mitochondrial 1C pathway in general, and MTHFD2 in particular, is critical for NADPH and nucleotide generation [34,43]. In addition, MTHFD2 is upregulated by oncogenic signalling, including mTOR activation and MYCN amplification in neuroblastoma, and is essential for metabolic reprogramming induced by these pro-oncogenic signalling proteins [29,31]. It is therefore not surprising that MTHFD2 is a drug target in cancer [44,45]. Here, we found that HSPD1 is co-expressed with MTHFD2, essential for maintaining MTHFD2 levels and that HSPD1 KD leads to reduced SAM levels and increased ROS. These findings suggest that HSPD1 promotes the 1C pathway by folding MTHFD2, and possibly other 1C enzymes, and that targeting HSPD1 will be impactful in cases where reduced 1C output is clinically desirable. In our hands, HSPD1 KD in A549 cells did lead to reduced MTHFD2 but not to reduced tumour growth. This is in contrast to previous work showing HSPD1 or MTHFD2 depletion leads to reduced tumour growth in A549 xenografts [46,47]. We do not know why we did not observe reduced tumour growth in our experiments, and speculate that this may be owing to differences in KD efficiency.

It was previously shown that GroEL can fold an exogenous target independently of GroES and ATP, acting as a catalytic unfoldase [37]. It was also found that HSPD1 interacts with ClpP through its epical domain to promote mitochondrial functions and tumorigenicity in cancer cells [48]. Using MTHFD2 and recombinant HSPD1, we found that HSPD1 can fold an endogenous client independently of HSPE1. In accord, HSPD1 and HSPE1 fractionated differently in thermal proteome profiling following mitochondrial stress activation [49], indicating that they interact with distinct protein complexes in these conditions. These lines of evidence, combined with our finding that HSPD1 folds its endogenous client, MTHFD2, independently of HSPE1, raise the possibility that HSPE1 may also have acquired biological functions that are HSPD1-independent.

The pipeline developed here, which led to the identification of MTHFD2 as an HSPD1 client, suggests that other HSPD1-dependent proteins identified using SILAC could also be HSPD1 clients. For example, we identified a number of mitochondrial tRNA synthetases whose levels are reduced in mitochondria of HSPD1 KD cells, suggesting that HSPD1 will impact mitochondrial translation. Indeed, recent work shows that HSPD1 KD reduces mitochondrial translation in yeast and mammalian cells [21].

In summary, we found that MTHFD2 is an endogenous client of HSPD1 whose folding is independent of HSPE1, linking mitochondrial proteostasis to the 1C pathway.

## 4. Methods

Please see supplementary material for methods.

## Supplementary Material



## Data Availability

The mass spectrometry proteomics data have been deposited to the ProteomeXchange Consortium via the PRIDE [1] partner repository with the dataset identifier PXD066753”. Project Name: The mitochondrial chaperone HSPD1 folds MTHFD2 independently of its co-chaperone HSPE1. Project accession: PXD066753. Supplementary material is available online [50].

## Theme

One contribution of 20 to a Theo Murphy meeting issue ‘ProteostaSys: a systems view of proteostasis’.
